# The Prevalence of Overfat Adults and Children in the US

**DOI:** 10.3389/fpubh.2017.00290

**Published:** 2017-11-01

**Authors:** Philip B. Maffetone, Paul B. Laursen

**Affiliations:** ^1^Independent Researcher, Oracle, AZ, United States; ^2^Sports Performance Research Institute New Zealand (SPRINZ), AUT University, Auckland, New Zealand

**Keywords:** overfat, obesity, overweight, chronic disease, inflammation, insulin resistance

## Abstract

The overfat condition is defined as excess body fat sufficient to impair health. The problem exists in most overweight and obese individuals and can also occur in those who are normal-weight and non-obese, often due to excess abdominal fat. Despite previous indications that the prevalence overweight and obesity is leveling, these conditions are currently at their highest levels in US history. Our review estimated the number of overfat Americans at 91% for adults and 69% for children. The primary purpose of this review was to build upon previous estimations of overfat prevalence in developed countries by using new data from the Centers for Disease Control and Prevention to estimate the overfat prevalence in American adults (≥20 years) and children (2–19 years), and to expand the definition of overfat as excess body fat associated with at least one additional risk factor of impaired cardiometabolic or physical health. The secondary goals are to highlight the role of dietary sugar as a primary cause of the overfat pandemic and mention new data showing the increased prevalence of exercise that parallels the rising prevalence of overfat to further emphasize the secondary role exercise may play in fat loss. Current public health guidelines to address the overfat pandemic may require more emphasis on reducing the consumption of refined carbohydrates, including added sugars.

## Key Findings

We define overfat as excess body fat associated with at least one additional risk factor of impaired cardiometabolic or physical health. Overfat can occur in normal-weight and non-obese individuals, often due to excess abdominal fat.
In the US, 91% of adults and 69% of children are estimated to be overfat.
Despite previous indications that the prevalence of individuals categorized as being overweight and obese is leveling, their prevalence is currently at their highest rates in US history.

## Introduction

Recently, the global overfat pandemic was estimated to encompass between 62 and 76% of the world’s total population (1). This relatively new term, *overfat*, refers to an accumulation of excess body fat that becomes sufficient to impair health (1). Even more recently, levels of overfat adults and children in developed countries have been shown as being substantially higher than worldwide averages (2). For example, in the US, 86% of adults and 52% of children were estimated to be overfat (2). The data used to calculate these estimates were derived in part from those shown by Ng et al. (3) for estimated overweight and obesity rates in US adults (66%). More recent data published by the Centers for Disease Control and Prevention (CDC) for 2014 show that over 70% of American adults are overweight or obese (4, 5). Determination of these overweight and obese classifications are usually based on measures of body mass index (BMI), with overweight defined with a score of ≥25 to <30 kg/m^2^ and obesity as ≥30 kg/m^2^. However, while BMI can overestimate fat mass in certain populations, including Polynesians, African American, and elite strength athletes, because BMI is not a direct measure of body fat it can misclassify up to 50% or more patients with both increased body fat and its associated disease risk factors (6, 7).

Estimations of overfat adults and children are important for public health reasons. Excess body fat is associated with poor health, and the development of insulin resistance and low-grade systemic chronic inflammation (8, 9). These conditions can lead to various cardiovascular and metabolic (cardiometabolic) impairments such as dyslipidemia, increased blood glucose, and hypertension, raising the risks of chronic diseases such as Type 2 diabetes (10, 11), cardiovascular diseases (12, 13), and most cancers (14). Chronic inflammation has also been linked to neurodegenerative diseases, including the most common cause of dementia, Alzheimer’s disease (15, 16).

The burden of excessive body fat can begin at an early age (17, 18), even in gestation (19), with over 55% of obese children estimated to have nonalcoholic fatty liver disease (20). Children with excess body fat are likely to feed the overfat pandemic and are at increased risk of becoming overfat adults with chronic disease (21–25).

The primary purpose of this review was to build upon previous estimations of overfat prevalence in developed countries by using new data from the CDC to estimate the overfat prevalence in American adults (≥20 years) and children (2–19 years), and to expand the definition of overfat as excess body fat associated with at least one additional risk factor of impaired cardiometabolic or physical health. The secondary goals were to highlight the role of dietary sugar as a potential primary cause of the overfat pandemic and mention new data showing the increased prevalence of exercise that parallels the rising prevalence of overfat to further emphasize the secondary role exercise may play in fat loss.

## Quantifying Overfat

While overfat was recently defined as excess body fat that impairs health (1), in this article, we offer an expanded, quantitative description in relation to its associated cardiometabolic, physical performance, and other measurable health impairments. Overfat is both an excess amount of body fat, depicted by increased body fat percentage or waist circumference (WC), and cardiovascular, metabolic, or physical impairments caused by being overfat (26). These topics are detailed in this section and throughout this review.

Excess body fat contributes to cardiometabolic impairments, resulting in various downstream conditions such as chronic inflammation and insulin resistance, risk factors such as abnormal blood glucose, cholesterol, triglycerides, and blood pressure, and chronic diseases, including Type 2 diabetes, cardiovascular disease, cancer, and Alzheimer’s disease (1). Increased body fat mass also raises the risk of mortality (27–29) and shares direct links to gallbladder disease, gout, pulmonary diseases, and sleep apnea (30), while the absence of excess body fatness lowers the risk of most cancers (14).

Physical performance impairments associated with excess adiposity include musculoskeletal disorders, such as lower-back and other pain syndromes, reduced work productivity in forms such as absenteeism and disability, as well as lower quality of life (31), and include locomotive disorders such as knee and hip osteoarthritis, lumbar spondylosis, and osteoporosis (32). Performance was also assessed in normal-weight obese children aged 3–6 years, who showed significantly worse levels of fundamental motor skills compared to their normal-weight non-obese counterparts (33). As fundamental motor skills in early life play a crucial role in physical, cognitive, and social development (34), childhood adiposity may indirectly exacerbate physical performance impairments into adulthood.

**Figure 1:**
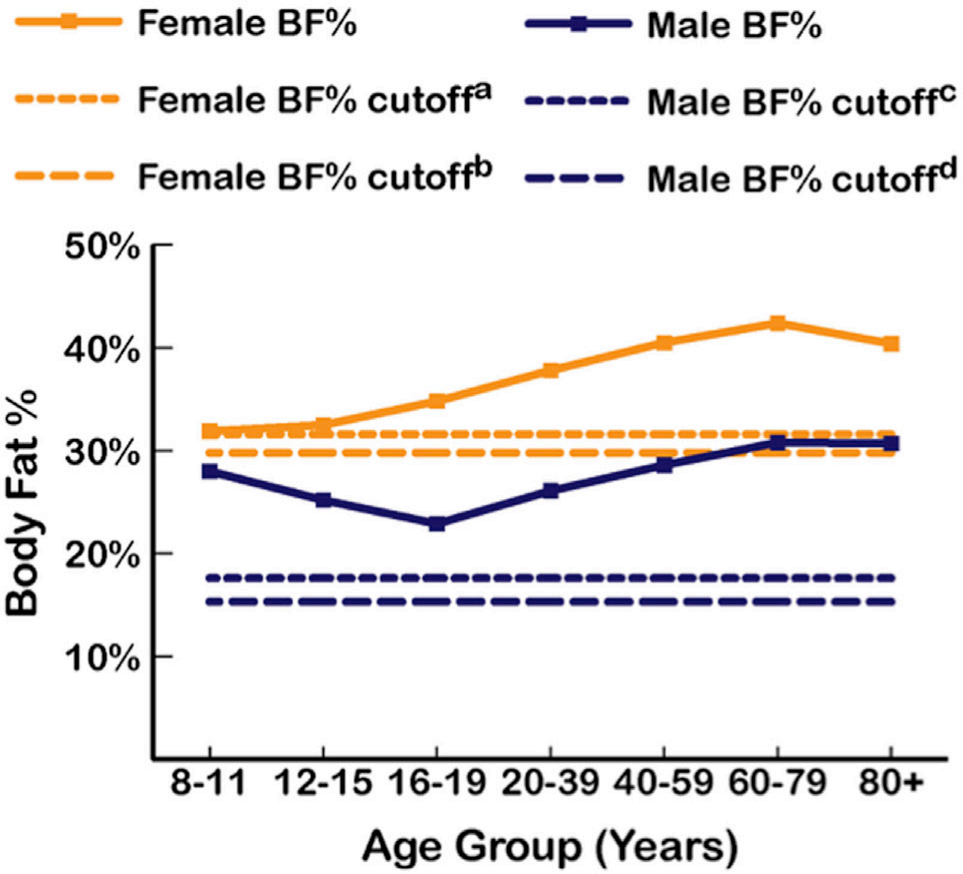
Mean percent body fat as dual-energy X-ray measurements from the 1999 to 2004 National Health and Nutrition Examination Survey (NHANES) by age group and sex (43), with body fat (BF) cutoffs from Lohman et al. (38) and Shea et al. (42). ^a^31.6% BF (Lohman et al.). ^b^29.8% BF (Shea et al.). ^c^17.6% BF (Lohman et al.). ^d^15.3% BF (Shea et al.).

As BMI often misclassifies body fatness, reductions of misclassification rates can be improved using dual energy X-ray absorptiometry (DXA) to directly measure body fat percentage (35). DXA is one of the most accurate and precise methods of assessing adiposity (36). While there is no consensus on how to define excess body fat percentage (37), Lohman’s criteria of suggested cutoffs >17.6% for males and >31.6% for females is widely accepted in body composition research (38–41). However, measurable health impairments associated with ≥2 cardiometabolic abnormalities were found at DXA-derived body fat levels >15.3% in men and >29.8% in women (42). DXA-derived body fat levels from the 1999 to 2004 National Health and Nutrition Examination Survey (NHANES) using Lohman’s criteria is shown in Figure 1, which shows mean body fat for men was 28%, with the cutoff of 17.6% representing approximately the 5th percentile (17.4%), and in women, mean body fat was 39.9%, with 31.6% between the 10th and 15th percentile (43).

The overfat condition can also be evaluated indirectly using waist measures, which, like BMI, do not directly estimate body fat percentage. Epidemiologic studies have demonstrated that increased abdominal fat, assessed through WC and waist-to-height ratio (WHtR), can predict adiposity-related risk (37). WHtR was more strongly related to cardiometabolic risk factors than BMI (29, 44). In normal-weight non-obese children with a commonly used cutoff of WHtR ≥ 0.5, over 55% had one to three cardiometabolic health risk factors associated with increases in WC, triglycerides, and blood pressure (45). Adults from the NHANES, 1999–2000 cohort showed that 86% of adults with abdominal obesity [defined as WC in men ≥ 102 cm (40″), in women ≥88 cm (35″)] had at least one other cardiometabolic risk factor (46).

Based on these observations, we summarize the definition of overfat as excess adiposity measured accurately (directly, such as by DXA, or indirectly, such as by WHtR) combined with at least one additional measurable risk factor of impaired cardiometabolic or physical health.

## Subcategories of Overfat

We have previously described overfat as a condition of excess body fat with two subcategories that include: (1) those who are overweight and/or obese with excess body fat and (2) normal-weight and non-obese individuals with excess body fat, including those with abdominal obesity and sarcopenic obesity (1, 2). Collectively, both subcategories are prevalent in adults and children, with the exception of the sarcopenic obesity condition, which is prevalent only in adults. The prevalence of the population who are overweight and obese has been well described in the literature and represents the largest subcategory of the overfat condition in the US and worldwide (1, 2).

### Overweight and Obesity

**Figure 2:**
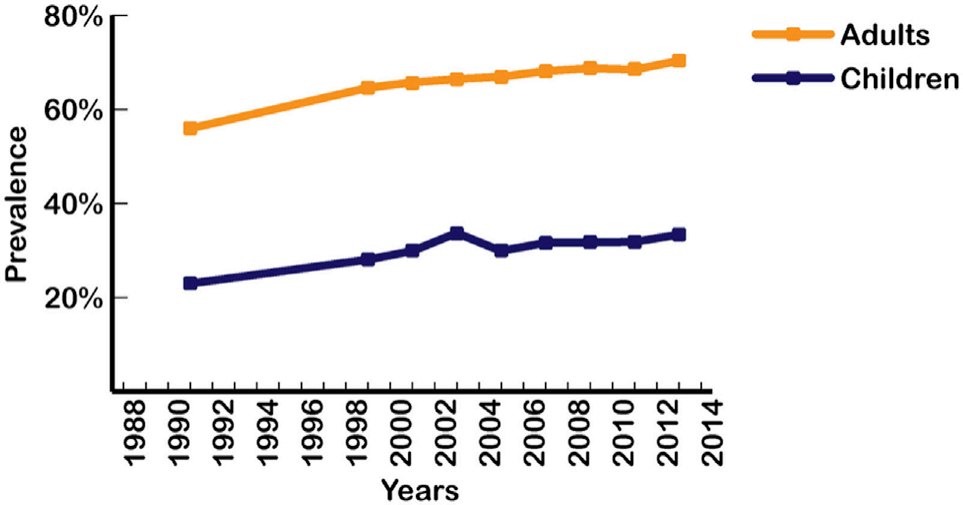
Prevalence of overweight and obesity in US adults and children from 1988 to 2014 (4, 5). Data points represent the middle year of each interval surveyed for adults and children. Intervals from left to right: 1988–1994; 1999–2000; 2001–2002; 2003–2004; 2005–2006; 2007–2008; 2009–2010; 2011–2012; 2013–2014. Based on crude estimates.

Increases in the prevalence of various subcategories of overfat have been shown to have temporary, separate periods of leveling-off at different points over the past three decades (47–49). Figure 2 shows trends in overweight and obesity classifications in Americans between 1988 and 1994 through to 2013–2014 reporting periods (crude data) (5), with both conditions currently at their highest levels in US history.

### Normal-Weight Non-Obese

Since the early observations of Ruderman et al. (50), the list of associated disorders clustered into the normal-weight non-obese overfat populations has grown to include insulin resistance and chronic inflammation, dyslipidemia, elevated fasting glucose, and hypertension, and a variety of chronic illnesses that include cardiovascular diseases, Type 2 diabetes, Alzheimer’s disease, and cancer (1, 2). These individuals are sometimes referred to as *metabolically obese normal weight*, or as *normal weight obese* (37, 51–53). It has been estimated that 20% of normal-weight non-obese adults may be metabolically obese normal weight (54), with 24% of normal-weight US adults (BMI < 25) considered metabolically abnormal with an elevated risk of chronic diseases typically associated with elevated BMI (55) and up to 40% at risk of developing metabolic syndrome (56).

The metabolically healthy obese (MHO) adult (BMI > 30) has also been recognized, with a prevalence of 7% worldwide (54). These subjects tend to be younger (<40) and, in the long term (>10 years) accumulate various cardiometabolic risk factors and diseases (57), and physical impairment (32). MHO individuals are still considered overfat (2, 37).

In children, studies that measured body fat percentage and the WHtR may each represent reasonable methods of estimating the population of normal-weight non-obese with excess body fat, including those with BMI < 25 and those with abdominal obesity (45, 58).

Two overfat conditions found in normal-weight non-obese individuals include abdominal obesity and sarcopenic obesity.

#### Abdominal Obesity

Excess body fat in the abdominal area includes both visceral adipose tissue and, separately, subcutaneous adipose tissue. Excess visceral fat is associated with more adverse risk factor profiles than subcutaneous fat, including its association with insulin resistance, dyslipidemia, and atherosclerosis (59–61). The excess accumulation of visceral fat is increasing among overweight and obese, as well as normal-weight non-obese individuals (48). The potential health risks of abdominal obesity are more pronounced than those due to excess body fat in other regions of the body (62). The recent rise in the prevalence of abdominal obesity in the US, which includes increases in WC, is found in both adults and children (45, 49). Because the problem can occur in non-obese individuals, this condition has been referred to as *abdominal overfat* (2).

#### Sarcopenic Obesity

Defined as a progressive loss of Type 2 fast-twitch muscle fibers and strength with aging, sarcopenic obesity has a prevalence of up to 50% in those >80 years (63). Sarcopenia can coincide with an accumulation of fat within existing muscle, with the combination of higher body fat and sarcopenia being termed *sarcopenic obesity* (64, 65). This condition occurs in those who are usually not obese and may be more appropriately called *sarcopenic overfat* (2).

## Overfat Adults and Children

To estimate the prevalence of overfat adults and children in the US, we combined the populations of overweight, obese, and normal-weight individuals with increased health risks (MONW). In adults, recent crude data estimated overweight and obese prevalence in the US at 70.7% (5). Metabolically obese normal weight prevalence has been estimated between 5 and 45%, with variations due to ethnicity, sample size effects, differences in MONW definition, social and demographic factors, and others (66, 67). The relatively recent prevalence of MONW adult populations in North America and Europe that included both genders and a wide range of ages have been estimated by various authors to be between 24 and 36% (55, 68–72). However, we feel the meta-analysis by Wang et al., which showed the overall prevalence of MONW adults in the general population worldwide at 20% (95% CI 16.54–23.94), was an appropriate estimate for the US population, and was previously used for estimating overfat prevalence in developed countries (2). Wang et al. (54) included publications between 2006 and 2013 with a wide range of populations including Europeans (individual countries and mixed populations), North Americans (US and Canada), and Asians (including Chinese, Indian, and Korean). Combining the categories of overweight, obese, and MONW US adults brings the prevalence of overfat in the US to 90.7%.

**Figure 3:**
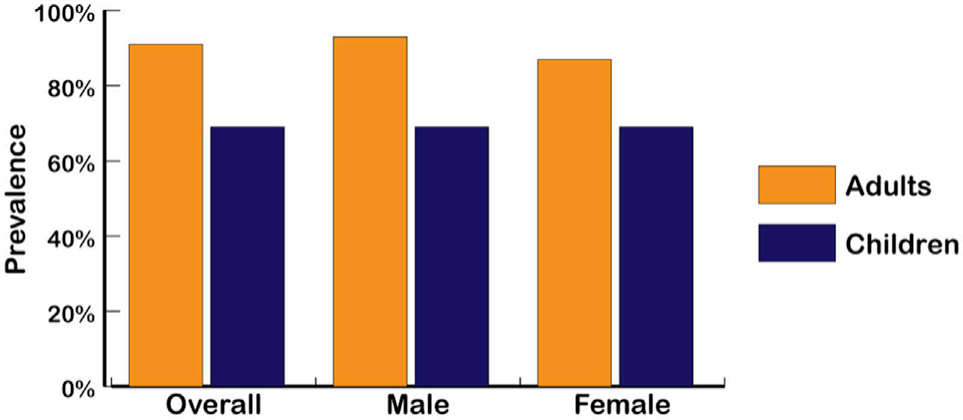
Overfat prevalence of US male and female adults and children (2013–14) (4, 5). Overfat defined as excess body fat associated with at least one additional risk factor of impaired cardiometabolic or physical health. Overfat can occur in normal-weight and non-obese individuals, often due to excess abdominal fat.

**Figure 4:**
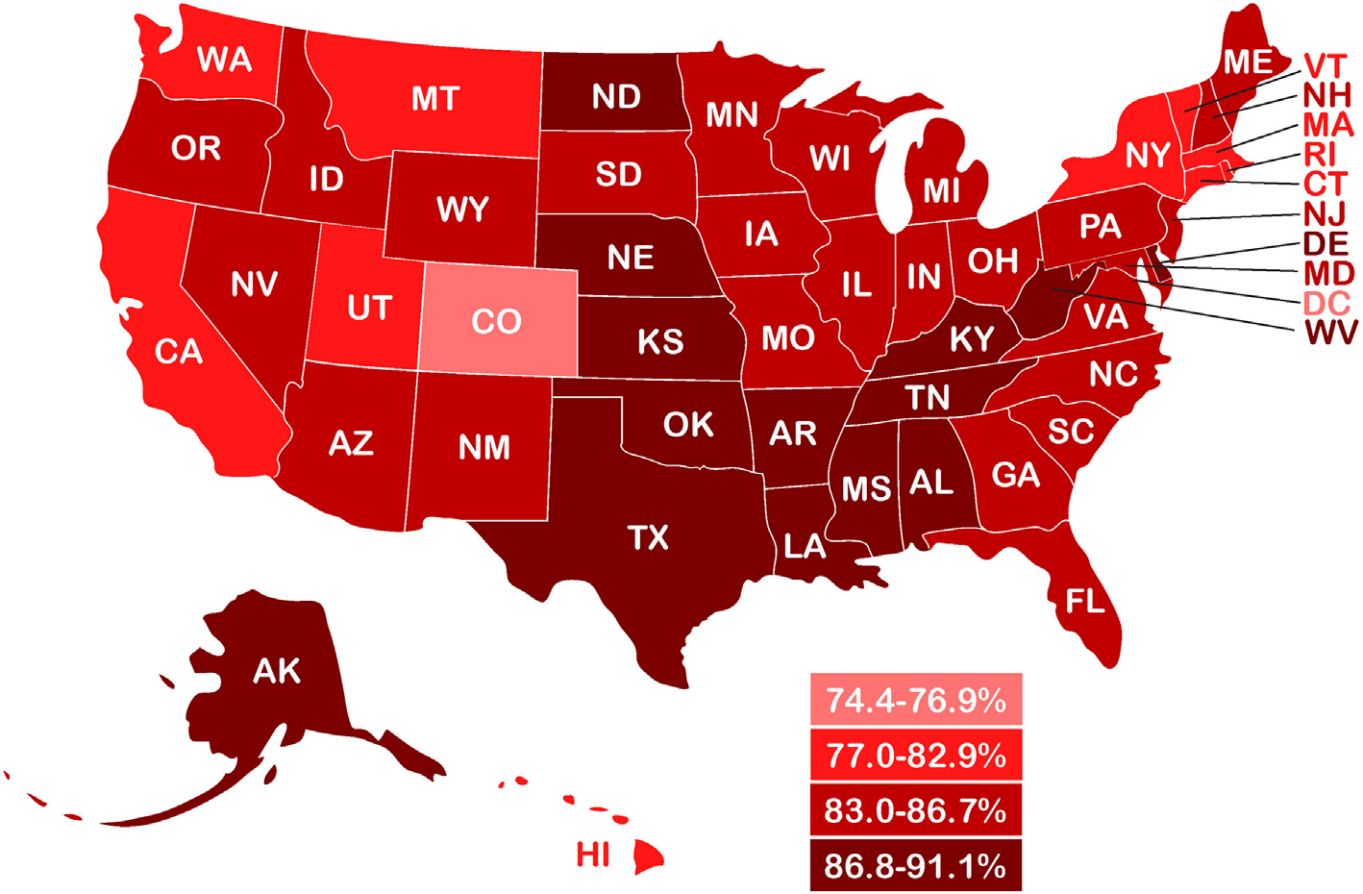
State-by-state prevalence of overfat adults (2013–14) based on reported prevalence of overweight and obesity (2, 74).

In children, as in adults, a wide range of normal weight high body fat conditions has been found in various populations. Mokha et al. (58) showed that in normal-weight non-obese children with a WHtR ≥ 0.5, over 55% had one to three cardiometabolic health risk factors associated with increases in WC, triglycerides, and blood pressure. In a Caucasian population of high school students, Olafsdottir et al. found 42% were normal-weight obese, defined as normal BMI with DXA body fat >17.6% in males and >31.6% in females, and with one or more risk factors for metabolic syndrome (39). Griffiths et al. (73) showed that 17.6% of normal-weight non-obese boys and 17.3% of normal-weight non-obese girls who exceeded a WHtR of 0.5 were considered “at risk,” as compared with children who were overweight or obese and above the 85th percentile, and Flegal et al. (45) estimated the prevalence of DXA-based adiposity in children with normal BMI below the 85th percentile and found that 35% within the 65th percentile cutoff and 24% within the 70th percentile cutoff had high adiposity. The WHtR-based study of Griffiths et al. (UK children) and the DXA-based data from Flegal et al. (US children) produced an average of 22% of overfat children (both genders), a figure also used in our recent estimates of children in developed countries (2). Combined with recent rates of US overweight and obese children aged 2–19 years of 46.9% (5), we estimated the overfat prevalence of US children to be 68.9%. Figure 3 shows the prevalence of overfat adults and children in the US, with State-by-State rates of overfat, which follow overweight and obese levels, shown in Figure 4.

## Health Effects of Excess Body Fat

While the health consequences of being overweight or obese are widely known, it is the specific condition of excess body fat that is associated with various disease risk factors, chronic illness, increased morbidity and mortality, and reduced quality of life. The problems associated with the rise in prevalence of overweight and obese classifications alone have replaced the longstanding problems of undernutrition and infectious diseases (30). Moreover, the overfat pandemic, with its potential downstream conditions, has created a major global economic burden (2). In a single year (2014) in the US, for example, total health-care costs climbed to $3.2 trillion US dollars (75). Overfat-related cardiometablic and physical impairments could be a substantial portion of these annual costs.

### Body Fat Measures

Various research methods have been used to quantify body fat levels in humans, including bioelectrical impedance and hydrostatic plethysmography, with DXA being one of the most accurate and precise methods currently accessible (36). Epidemiologic studies have also demonstrated that abdominal fat distribution, assessed through WC, waist-to-hip ratio and WHtR measurements, can be useful in the clinical assessment of adiposity-related risk (37), although, like BMI, these measures do not accurately estimate body fat percentage. Of these, the WHtR may be the most convenient, inexpensive, and valuable clinical indicator of health and overfat risk for use in all ethnic groups of adults and children (76, 77). A simple recommendation is that the WC measurement should be less than half of a person’s height (2).

While recognition of the staggering prevalence of the overfat pandemic is a key step in addressing this public health problem, it is difficult to discuss the issue without at least briefly addressing its possible causes.

## Lifestyle Influence on Body Fat

In addition to genetic influences, two lifestyle factors, physical activity, and dietary sugar, can play key roles in the development of excess body fat and are reviewed here.

### Physical Activity

**Figure 5:**
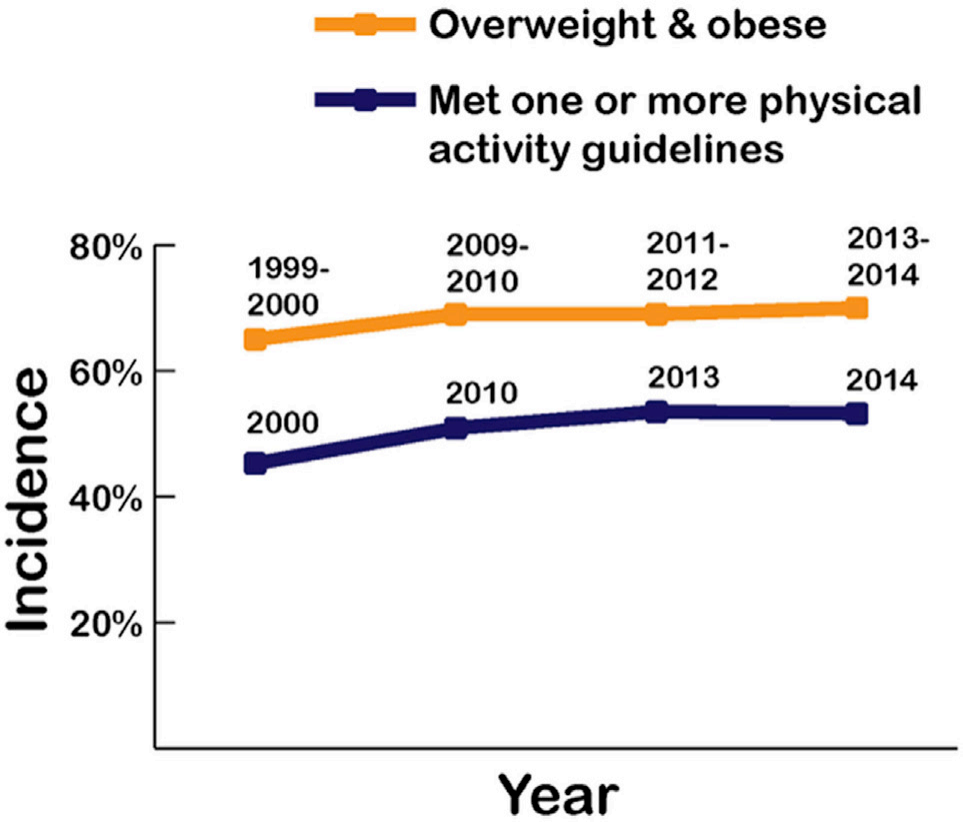
Recorded prevalence of being classified as overweight and obese alongside physical activity rates in US adults from 1999 to 2014 (4, 5).

More than half of US adults meet the federal 2008 *Physical Activity Guidelines for Americans* and regularly exercise using either aerobic or muscle-strengthening exercise, with an increase shown rising from 44% in 1998 to almost 52% in 2014 (5). Those meeting these guidelines for *both* aerobic activity and muscle-strengthening exercise also increased from about 14% in 1998 to 21% in 2014. However, as shown in Figure 5, the recorded rates for adults being either overweight or obese rose to over 70.7% during a similar period, reflecting the overfat prevalence increase from 75 to over 90% (Figure 3).

Whether these exercise recommendations are adequate or accurate is not known. However, physical activity and exercise is expected to increase energy expenditure and whole body fat oxidation, with a sedentary lifestyle decreasing insulin sensitivity and fat oxidation, potentially increasing stored body fat (78–82). This includes elite athletes and those in active military, who can also have excess body fat (83–86). In healthy adults, high glycemic foods can also reduce overall energy expenditure by nearly 50% compared with isoenergetic meals containing whole foods (87).

While some authors have placed a strong emphasis on the importance of physical activity for reducing excess body fat (88), others feel that physical activity plays a lesser role in the development of the overfat pandemic (89). Indeed, Luke and Cooper (90) argue that despite dramatic increases in obesity rates over the past 30 years, physical activity levels in the Western population have not reduced and changed little. Swinburn et al. (91) showed that increased energy intake appears to be more than sufficient to explain weight gain in the US population, contending that the simultaneous increase in most country’s obesity rates appear driven mainly by a shift toward more processed, affordable, and effectively marketed food than ever before. Finally, Basu et al. (92) showed that differences in sugar availability could explain variations in diabetes prevalence at a population level that is not explained by physical activity or being overfat. Clearly, these opposing trends, that is, the rise in the rates of overfat despite increases in physical activity are at odds with the belief that an increase in physical activity (energy expenditure) is the solution to the overfat pandemic.

### Dietary Sugar

The largest contributing lifestyle factor to overfat would appear to be excess sugar consumption. Indeed, excess sugar in foods plays a major role in the cause of the rising rates of overweight and obesity classifications, including the monosaccharides, glucose and fructose, and the disaccharides maltose and sucrose (93). As sugar-sweetened beverages are the single largest source of added sugar and the top source of energy intake in the US diet (94), their increased consumption among adults and children may be a primary contributor to the overfat pandemic (95–98). The consumption of sugar-sweetened beverages is also strongly associated with chronic illness (99).

The recommendations from the Institute of Medicine, the American Heart Association, the Obesity Society, and many other organizations is to reduce consumption of soft-drinks for children and adults (100), with the US Dietary Guidelines Advisory Committee recommending that added sugars constitute <10% of total calories per day (101). However, these recommendations have not yet been effectively implemented.

In addition to sugar-sweetened beverages, high glycemic diets, those containing foods with added sugar and other refined carbohydrates, can contribute to insulin resistance and chronic inflammation, and to the development of Type 2 diabetes, dyslipidemia, nonalcoholic liver disease, cardiovascular disease, and other chronic illness in adults and children (18, 102–104). Average daily consumption of added sugar for men (335 calories) and women (230 calories) exceed recommendations of the American Heart Association of 150 and 100 calories, respectively (105). This is particularly true of refined sugar and other refined carbohydrate consumption beginning very early in life, which occurs despite current evidence suggesting that children also limit added sugar intake to <10% of total energy (106).

It is well established that early life exposure to excess sugars (other than lactose) could influence taste preferences, satiety, and health (107). In animal studies, maternal exposure to sugar during pregnancy and lactation have shown long-lasting overfat promoting effects in their offspring (108). Walker and Goran (109) found that 74% of 100 selected test samples of infant formulas, baby and children’s cereals, and other retail food items analyzed in a laboratory contained ≥20% of total calories per serving, mostly from added sugars. Of 20 yogurt products tested, the mean percent of sugar from added sugar was 63% (37% on average was naturally occurring). The popularity of packaged foods represents one problem of hidden sugars, as nutrition databases and, therefore, nutrition labels may not accurately reflect true sugar content. A number of baby food products tested by Walker and Goran (109) indicated no added sugar in their ingredients, when in actuality they contained high sugar content.

The effects of excess dietary sugar can influence cardiometabolic health rapidly. For example, reducing consumption of added sugars in the diet, without reducing calories or losing body mass, reversed a cluster of chronic metabolic diseases in children, including high cholesterol, hypertension, and nonalcoholic liver disease, within only 9 days (110, 111).

**Figure 6:**
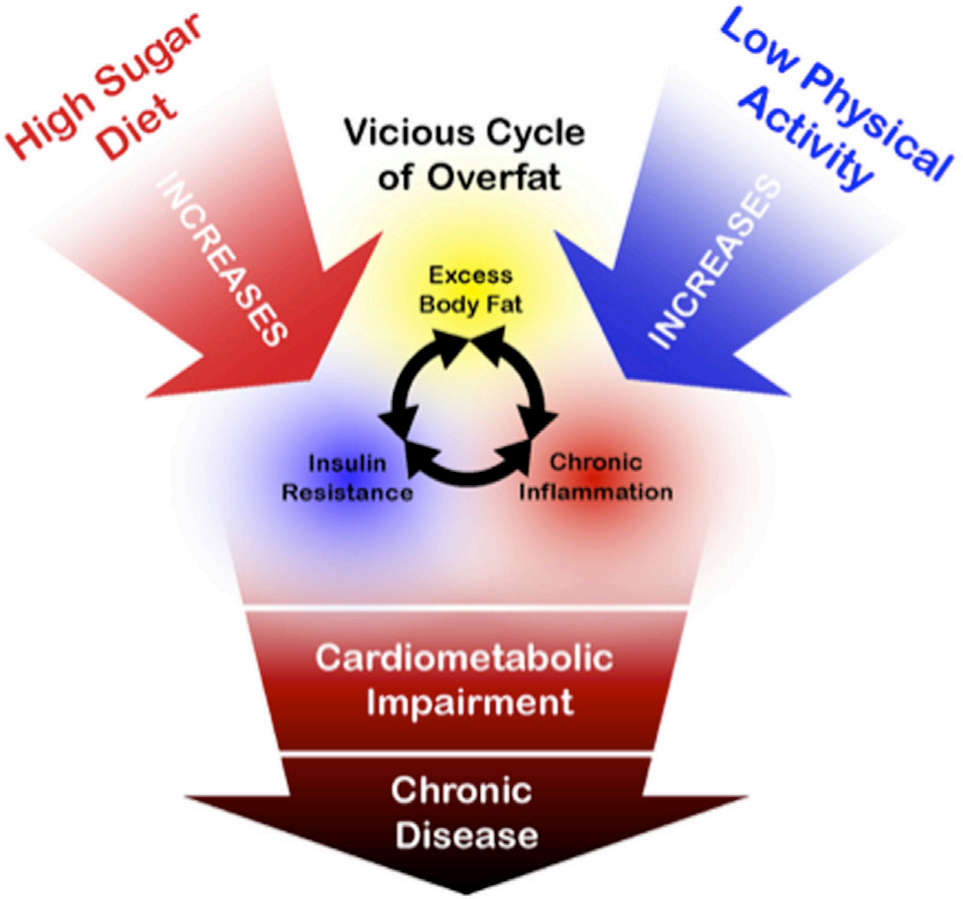
Schematic drawing illustrating general relationships between overfat and its downstream conditions, alongside the primary influence of a high sugar diet. While physical activity is of importance for a number of health-related factors, its influence on reducing the overfat occurrence is marginal by comparison with the lowering of sugar content in the diet (89–91, 94–106).

While physical activity has many known health benefits and should continue to be recommended, as depicted in Figure 6, the above arguments suggest that current public health guidelines addressing the overfat pandemic require far more emphasis on reducing the consumption of refined carbohydrates, including added sugars.

An important focus of this review was to emphasize the extensiveness of the overfat pandemic as a primary public health problem deserving of more clinical attention, not unlike The National Cholesterol Education Program’s Adult Treatment Panel III report that states obesity be the primary target of intervention for metabolic syndrome (26). Indeed, the prevalence of overfat is much greater, and the need for further research and consensus to build on the overfat definition presented here is obvious.

## Conclusion

The number of overfat Americans continues to rise, with estimations of 91% in adults and 69% in children, the highest levels in US history. Recognition of the overfat pandemic is a key step in initiating its reversal and prevention. Because overfat prevalence continued rising during periods of increased exercise rates, current public health guidelines to address the overfat pandemic may require more emphasis on food, especially in reducing consumption of excess refined carbohydrates, including added sugars.

## Author Contributions

PM—originator of ideas, data collection, analysis, critical thinking, and writing of the manuscript. PL—contributed to ideas, critical thinking, and writing of the manuscript.

## Conflict of Interest Statement

The authors declare that the research was conducted in the absence of any commercial or financial relationships that could be construed as a potential conflict of interest. PM is an independent clinical consultant, writes articles and books that include the topics presented herein, and has a business website pertaining to health and fitness (www.philmaffetone.com). IR-D works for a health and fitness company. PL is an independent consultant, writes articles and books, and has a website pertaining to performance, health, and longevity (www.plewsandprof.com).

## References

[1] Maffetone PB Rivera-Dominguez I Laursen PB. Overfat and underfat: new terms and definitions long overdue. Front Public Health (2017) 4:279. 10.3389/fpubh.2016.00279 28097119 PMC5206235

[2] Maffetone PB Rivera-Dominguez I Laursen PB Overfat adults and children in developed countries: the public health importance of identifying excess body fat. Front Public Health (2017) 5:190 10.3389/fpubh.2017.00190 28791284 PMC5523552

[3] Ng M Fleming T Robinson M Thomson B Graetz N Margono C Global, regional, and national prevalence of overweight and obesity in children and adults during 1980–2013: a systematic analysis for the Global Burden of Disease Study 2013. Lancet (2014) 384(9945):766–81. 10.1016/S0140-6736(14)60460-8 24880830 PMC4624264

[4] Centers for Disease Control and Prevention (CDC). Disability and Risk Factors: Obesity and Overweight. Centers for Disease Control and Prevention (2017). Available from: https://www.cdc.gov/nchs/fastats/obesity-overweight.htm

[5] National Center for Health Statistics (NCHS). Health, United States: With Special Feature on Racial and Ethnic Disparities. Hyattsville, MD: Centers for Disease Control (2016). Available from: https://www.cdc.gov/nchs/data/hus/hus15.pdf - 053 27308685

[6] Romero-Corral A Somers VK Sierra-Johnson J Thomas RJ Collazo-Clavell M Korinek J Accuracy of body mass index in diagnosing obesity in the adult general population. Int J Obes (2008) 32(6):959–66. 10.1038/ijo.2008.11 18283284 PMC2877506

[7] Okorodudu D Jumean M Montori VM Romero-Corral A Somers V Erwin P Diagnostic performance of body mass index to identify obesity as defined by body adiposity: a systematic review and meta-analysis. Int J Obes (2010) 34(5):791–9. 10.1038/ijo.2010.5 20125098

[8] Giorgino F Laviola L Eriksson JW. Regional differences of insulin action in adipose tissue: insights from in vivo and in vitro studies. Acta Physiol Scand (2005) 183(1):13–30. 10.1111/j.1365-201X.2004.01385.x 15654917

[9] Park MH Kim DH Lee EK Kim ND Im DS Lee J Age-related inflammation and insulin resistance: a review of their intricate interdependency. Arch Pharm Res (2014) 37(12):1507–14. 10.1007/s12272-014-0474-6 25239110 PMC4246128

[10] Pradhan AD Manson JE Rifai N Buring JE Ridker PM. C-reactive protein, interleukin 6, and risk of developing type 2 diabetes mellitus. JAMA (2001) 286(3):327–34. 10.1001/jama.286.3.327 11466099

[11] Donath MY Shoelson SE. Type 2 diabetes as an inflammatory disease. Nat Rev Immunol (2011) 11(2):98–107. 10.1038/nri2925 21233852

[12] Romero-Corral A Somers VK Sierra-Johnson J Korenfeld Y Boarin S Korinek J Normal weight obesity: a risk factor for cardiometabolic dysregulation and cardiovascular mortality. Eur Heart J (2010) 31(6):737–46. 10.1093/eurheartj/ehp487 19933515 PMC2838679

[13] Poirier P Giles TD Bray GA Hong Y Stern JS Pi-Sunyer FX Obesity and cardiovascular disease: pathophysiology, evaluation, and effect of weight loss. Circulation (2006) 113(6):898–918. 10.1161/01.ATV.0000216787.85457.f3 16380542

[14] Lauby-Secretan B Scoccianti C Loomis D Grosse Y Bianchini F Straif K Body fatness and cancer—viewpoint of the IARC working group. N Engl J Med (2016) 375(8):794–8. 10.1056/NEJMsr1606602 27557308 PMC6754861

[15] Rochfort KD Cummins PM The blood–brain barrier endothelium: a target for pro-inflammatory cytokines. Biochem Soc Trans (2015) 43(4):702–6. 10.1042/BST20140319 26551716

[16] Minter MR Taylor JM Crack PJ. The contribution of neuroinflammation to amyloid toxicity in Alzheimer’s disease. J Neurochem (2016) 136(3):457–74. 10.1111/jnc.13411 26509334

[17] Ip EH Leng X Zhang Q Schwartz R Chen S-H Dai S Risk profiles of lipids, blood pressure, and anthropometric measures in childhood and adolescence: project heartBeat! BMC Obes (2016) 3(1):9. 10.1186/s40608-016-0090-8 26929822 PMC4758172

[18] Weiss R Bremer AA Lustig RH. What is metabolic syndrome, and why are children getting it? Ann N Y Acad Sci (2013) 1281(1):123–40. 10.1111/nyas.12030 23356701 PMC3715098

[19] Catalano PM Presley L Minium J Hauguel-de Mouzon S Fetuses of obese mothers develop insulin resistance in utero. Diabetes Care (2009) 32(6):1076–80. 10.2337/dc08-2077 19460915 PMC2681036

[20] Zou CC Liang L Fang H Fu JF Zhao ZY. Serum adiponectin, resistin levels and non-alcoholic fatty liver disease in obese children. Endocr J (2005) 52(5):519–24. 10.1507/endocrj.52.519 16284427

[21] Park M Falconer C Viner R Kinra S. The impact of childhood obesity on morbidity and mortality in adulthood: a systematic review. Obes Rev (2012) 13(11):985–1000. 10.1111/j.1467-789X.2012.01015.x 22731928

[22] Krassas G Tzotzas T Do obese children become obese adults: childhood predictors of adult disease. Pediatr Endocrinol Rev (2004) Suppl 3:455–9. 16444174

[23] Singh AS Mulder C Twisk JW Van Mechelen W Chinapaw MJ. Tracking of childhood overweight into adulthood: a systematic review of the literature. Obes Rev (2008) 9(5):474–88. 10.1111/j.1467-789X.2008.00475.x 18331423

[24] Schubert CM Sun SS Burns TL Morrison JA Huang TT-K Predictive ability of childhood metabolic components for adult metabolic syndrome and type 2 diabetes. J Pediatr (2009) 155(3):S6.e1–7. 10.1016/j.jpeds.2009.04.048 PMC3777811 19732565

[25] Baker JL Olsen LW Sorensen TI Childhood body-mass index and the risk of coronary heart disease in adulthood. J Vasc Surg (2008) 47(4):893–4. 10.1016/j.jvs.2008.02.015

[26] Grundy SM Brewer HB Cleeman JI Smith SC Lenfant C Definition of metabolic syndrome: report of the National Heart, Lung, and Blood Institute/American Heart Association conference on scientific issues related to definition. Circulation (2004) 109(3):433 10.1161/01.CIR.0000111245.75752.C6 14744958

[27] The Global Burden of Metabolic Risk Factors for Chronic Diseases Collaboration Lu Y Hajifathalian K Ezzati M Woodward M Rimm EB Metabolic mediators of the effects of body-mass index, overweight, and obesity on coronary heart disease and stroke: a pooled analysis of 97 prospective cohorts with 1 8 million participants. Lancet (2014) 383(9921):970–83. 10.1016/S0140-6736(13)61836-X 24269108 PMC3959199

[28] Allison DB Zhu S Plankey M Faith MS Heo M. Differential associations of body mass index and adiposity with all-cause mortality among men in the first and second National Health and Nutrition Examination Surveys (NHANES I and NHANES II) follow-up studies. Int J Obes (2002) 26(3):410. 10.1038/sj.ijo.0801925 11896498

[29] Zhu S Heo M Plankey M Faith MS Allison DB. Associations of body mass index and anthropometric indicators of fat mass and fat free mass with all-cause mortality among women in the first and second National Health and Nutrition Examination Surveys follow-up studies. Ann Epidemiol (2003) 13(4):286–93. 10.1016/S1047-2797(02)00417-9 12684196

[30] WHO. Obesity: preventing and managing the global epidemic. Report of a WHO consultation. World Health Organ Tech Rep Ser (2000) 894:i–xii, 1–253. 10.1017/S0021932003245508 11234459

[31] Seidell JC Kahn HS Williamson DF Lissner L Valdez R Report from a Centers for Disease Control and Prevention Workshop on use of adult anthropometry for public health and primary health care. Am J Clin Nutr (2001) 73:123–6. 11124761 10.1093/ajcn/73.1.123

[32] Yoshimura N Muraki S Nakamura K Tanaka S Epidemiology of the locomotive syndrome: the research on osteoarthritis/osteoporosis against disability study 2005–2015. Mod Rheumatol (2017) 27(1):1–7. 10.1080/14397595.2016.1226471 27538793

[33] Musalek M Kokstejn J Papez P Scheffler C Mumm R Czernitzki A Impact of normal weight obesity on fundamental motor skills in pre-school children aged 3 to 6 years. Anthropol Anz (2017) 74:203–12. 10.1127/anthranz/2017/0752 28765872

[34] Clark JE Metcalfe JS The mountain of motor development: a metaphor. In: Clark JE Humphrey JH, editors. Motor Development: Research and Reviews. (Vol. 2), Reston, VA: NASPE Pulications (2002). p. 163–90.

[35] Pedersen SD Astrup A Skovgaard I Reduction of misclassification rates of obesity by body mass index using dual-energy X-ray absorptiometry scans to improve subsequent prediction of per cent fat mass in a Caucasian population. Clin Obes (2011) 1(2–3):69–76. 10.1111/j.1758-8111.2011.00016.x 25585571

[36] Cornier M Després J Davis N Grossniklaus D Klein S Lamarche B Assessing adiposity: a scientific statement from the American Heart Association. Circulation (2011) 124(18):1996–2019. 10.1161/CIR.0b013e318233bc6a 21947291

[37] Oliveros E Somers VK Sochor O Goel K Lopez-Jimenez F. The concept of normal weight obesity. Prog Cardiovasc Dis (2014) 56(4):426–33. 10.1016/j.pcad.2013.10.003 24438734

[38] Lohman TG Houtkooper L Going SB Body fat measurement goes high-tech: not all are created equal. ACSMs Health Fit J (1997) 1(1):30–5.

[39] Olafsdottir AS Torfadottir JE Arngrimsson SA. Health behavior and metabolic risk factors associated with normal weight obesity in adolescents. PLoS One (2016) 11(8):e0161451. 10.1371/journal.pone.0161451 27560824 PMC4999227

[40] Hinriksdóttir G Tryggvadóttir Á Ólafsdóttir AS Arngrímsson SÁ Fatness but not fitness relative to the fat-free mass is related to C-reactive protein in 18 year-old adolescents. PLoS One (2015) 10(6):e0130597 10.1371/journal.pone.0130597 26075745 PMC4468067

[41] Evans E Rowe D Racette S Ross K McAuley E. Is the current BMI obesity classification appropriate for black and white postmenopausal women? Int J Obes (Lond) (2006) 30(5):837–43. 10.1038/sj.ijo.0803208 16418761

[42] Shea J King M Yi Y Gulliver W Sun G. Body fat percentage is associated with cardiometabolic dysregulation in BMI-defined normal weight subjects. Nutr Metab Cardiovasc Dis (2012) 22(9):741–7. 10.1016/j.numecd.2010.11.009 21215604

[43] Centers for Disease Control and Prevention (CDC). National Health and Nutrition Examination Survey [Internet]. National Health and Nutrition Examination Survey, 1999–2004. (2015). Available from: http://www.cdc.gov/nchs/nhanes.htm

[44] Hetherington-Rauth M Bea JW Lee VR Blew RM Funk J Lohman TG Comparison of direct measures of adiposity with indirect measures for assessing cardiometabolic risk factors in preadolescent girls. Nutr J (2017) 16(1):15. 10.1186/s12937-017-0236-7 28231807 PMC5324258

[45] Flegal KM Ogden CL Yanovski JA Freedman DS Shepherd JA Graubard BI High adiposity and high body mass index-for-age in US children and adolescents overall and by race-ethnic group. Am J Clin Nutr (2010) 91(4):1020–6. 10.3945/ajcn.2009.28589 20164313 PMC2844683

[46] Centers for Disease Control and Prevention (CDC). National Center for Health Statistics: National Health and Nutrition Examination Survey Data. Questionnaires, Datasets, and Related Documentation. (2016). Available from: http://www.cdc.gov/nchs/nhanes/nhanes_questionnaires.htm

[47] Flegal KM Kruszon-Moran D Carroll MD Fryar CD Ogden CL. Trends in obesity among adults in the United States, 2005 to 2014. JAMA (2016) 315(21):2284–91. 10.1001/jama.2016.6458 27272580 PMC11197437

[48] Ford ES Maynard LM Li C Trends in mean waist circumference and abdominal obesity among US adults, 1999–2012. JAMA (2014) 312(11):1151–3. 10.1001/jama.2014.8362 25226482 PMC4608432

[49] Xi B Mi J Zhao M Zhang T Jia C Li J Trends in abdominal obesity among US children and adolescents. Pediatrics (2014) 134(2):e334–9. 10.1542/peds.2014-0970 25049347

[50] Ruderman N Berchtold P Schneider S Obesity-associated disorders in normal-weight individuals: some speculations. Int J Obes (1981) 6:151–7. 6749721

[51] Ruderman N Chisholm D Pi-Sunyer FX Schneider S. The metabolically obese, normal-weight individual revisited. Diabetes (1998) 47(5):699–713. 10.2337/diabetes.47.5.699 9588440

[52] Hollenbeck C Reaven GM. Variations in insulin-stimulated glucose uptake in healthy individuals with normal glucose tolerance. J Clin Endocrinol Metab (1987) 64(6):1169–73. 10.1210/jcem-64-6-1169 3553221

[53] De Lorenzo A Martinoli R Vaia F Di Renzo L. Normal weight obese (NWO) women: an evaluation of a candidate new syndrome. Nutr Metab Cardiovasc Dis (2006) 16(8):513–23. 10.1016/j.numecd.2005.10.010 17126766

[54] Wang B Zhuang R Luo X Yin L Pang C Feng T Prevalence of metabolically healthy obese and metabolically obese but normal weight in adults worldwide: a meta-analysis. Horm Metab Res (2015) 47(11):839–45. 10.1055/s-0035-1559767 26340705

[55] Wildman RP Muntner P Reynolds K McGinn AP Rajpathak S Wylie-Rosett J The obese without cardiometabolic risk factor clustering and the normal weight with cardiometabolic risk factor clustering: prevalence and correlates of 2 phenotypes among the US population (NHANES 1999–2004). Arch Intern Med (2008) 168(15):1617–24. 10.1001/archinte.168.15.1617 18695075

[56] Lustig RH Schmidt LA Brindis CD Public health: the toxic truth about sugar. Nature (2012) 482(7383):27–9. 10.1038/482027a 22297952

[57] Meigs JB Wilson PW Fox CS Vasan RS Nathan DM Sullivan LM Body mass index, metabolic syndrome, and risk of type 2 diabetes or cardiovascular disease. J Clin Endocrinol Metab (2006) 91(8):2906–12. 10.1210/jc.2006-0594 16735483

[58] Mokha JS Srinivasan SR DasMahapatra P Fernandez C Chen W Xu J Utility of waist-to-height ratio in assessing the status of central obesity and related cardiometabolic risk profile among normal weight and overweight/obese children: the Bogalusa Heart Study. BMC Pediatr (2010) 10(1):73. 10.1186/1471-2431-10-73 20937123 PMC2964659

[59] Fox CS Massaro JM Hoffmann U Pou KM Maurovich-Horvat P Liu C-Y Abdominal visceral and subcutaneous adipose tissue compartments. Circulation (2007) 116(1):39–48. 10.1161/CIRCULATIONAHA.106.675355 17576866

[60] Despres J Lemieux S Lamarche B Prud’Homme D Moorjani S Brun L The insulin resistance-dyslipidemic syndrome: contribution of visceral obesity and therapeutic implications. Int J Obes Relat Metab Disord (1995) 19:S76. 7550542

[61] Scherzer R Shen W Bacchetti P Kotler D Lewis CE Shlipak MG Simple anthropometric measures correlate with metabolic risk indicators as strongly as magnetic resonance imaging-measured adipose tissue depots in both HIV-infected and control subjects. Am J Clin Nutr (2008) 87(6):1809–17. 18541572 10.1093/ajcn/87.6.1809 PMC2587301

[62] Flegal KM Carroll MD Kit BK Ogden CL. Prevalence of obesity and trends in the distribution of body mass index among US adults, 1999–2010. JAMA (2012) 307(5):491–7. 10.1001/jama.2012.39 22253363

[63] Ali S Garcia JM. Sarcopenia, cachexia and aging: diagnosis, mechanisms and therapeutic options – a mini-review. Gerontology (2014) 60(4):294–305. 10.1159/000356760 24731978 PMC4112511

[64] Sakuma K Yamaguchi A. Sarcopenic obesity and endocrinal adaptation with age. Int J Endocrinol (2013) 2013:204164. 10.1155/2013/204164 23690769 PMC3639625

[65] Muscaritoli M Anker S Argiles J Aversa Z Bauer J Biolo G Consensus definition of sarcopenia, cachexia and pre-cachexia: joint document elaborated by Special Interest Groups (SIG) “cachexia-anorexia in chronic wasting diseases” and “nutrition in geriatrics”. Clin Nutr (2010) 29(2):154–9. 10.1016/j.clnu.2009.12.004 20060626

[66] Ding C Chan Z Magkos F Lean, but not healthy: the ‘metabolically obese, normal-weight’ phenotype. Curr Opin Clin Nutr Metab Care (2016) 19(6):408–17. 10.1097/MCO.0000000000000317 27552473

[67] Heo M Faith MS Pietrobelli A Heymsfield SB Percentage of body fat cutoffs by sex, age, and race-ethnicity in the US adult population from NHANES 1999–2004. Am J Clin Nutr (2012) 95(3):594–602. 10.3945/ajcn.111.025171 22301924

[68] Lopez-Garcia E Guallar-Castillon P Leon-Muñoz L Rodriguez-Artalejo F. Prevalence and determinants of metabolically healthy obesity in Spain. Atherosclerosis (2013) 231(1):152–7. 10.1016/j.atherosclerosis.2013.09.003 24125427

[69] Shea JL Randell EW Sun G The prevalence of metabolically healthy obese subjects defined by BMI and dual-energy X-ray absorptiometry. Obesity (Silver Spring) (2011) 19(3):624–30. 10.1038/oby.2010.174 20706202

[70] Phillips CM Dillon C Harrington JM McCarthy VJ Kearney PM Fitzgerald AP Defining metabolically healthy obesity: role of dietary and lifestyle factors. PLoS One (2013) 8(10):e76188. 10.1371/journal.pone.0076188 24146838 PMC3798285

[71] Gomez-Huelgas R Narankiewicz D Villalobos A Wärnberg J Mancera-Romero J Cuesta A Prevalence of metabolically discordant phenotypes in a mediterranean population—the IMAP study. Endocr Pract (2013) 19(5):758–68. 10.4158/EP12355.OR 23757607

[72] Pajunen P Kotronen A Korpi-Hyövälti E Keinänen-Kiukaanniemi S Oksa H Niskanen L Metabolically healthy and unhealthy obesity phenotypes in the general population: the FIN-D2D Survey. BMC Public Health (2011) 11(1):754. 10.1186/1471-2458-11-754 21962038 PMC3198943

[73] Griffiths C Gately P Marchant PR Cooke CB. Cross-sectional comparisons of BMI and waist circumference in British children: mixed public health messages. Obesity (2012) 20(6):1258–60. 10.1038/oby.2011.294 21959348

[74] Centers for Disease Control and Prevention (CDC). Behavioral Risk Factor Surveillance System (BRFSS). Chronic Disease Indicators (CDI). (2015). Available from: https://www.cdc.gov/cdi/

[75] Martin AB Hartman M Benson J Catlin A Team NHEA. National health spending in 2014: faster growth driven by coverage expansion and prescription drug spending. Health Aff (2016) 35(1):150–60. 10.1377/hlthaff.2015.1194 26631494

[76] Ashwell M Hsieh SD. Six reasons why the waist-to-height ratio is a rapid and effective global indicator for health risks of obesity and how its use could simplify the international public health message on obesity. Int J Food Sci Nutr (2005) 56(5):303–7. 10.1080/09637480500195066 16236591

[77] Savva S Tornaritis M Savva M Kourides Y Panagi A Silikiotou N Waist circumference and waist-to-height ratio are better predictors of cardiovascular disease risk factors in children than body mass index. Int J Obes (2000) 24(11):1453 10.1038/sj.ijo.0801401 11126342

[78] Rimbert V Boirie Y Bedu M Hocquette J-F Ritz P Morio B Muscle fat oxidative capacity is not impaired by age but by physical inactivity: association with insulin sensitivity. FASEB J (2004) 18(6):737–9. 10.1096/fj.03-1104fje 14977873

[79] Smorawiński J Nazar K Kaciuba-Uscilko H Kamińska E Cybulski G Kodrzycka A Effects of 3-day bed rest on physiological responses to graded exercise in athletes and sedentary men. J Appl Physiol (2001) 91(1):249–57. 11408437 10.1152/jappl.2001.91.1.249

[80] Sidossis LS Stuart CA Shulman GI Lopaschuk GD Wolfe RR. Glucose plus insulin regulate fat oxidation by controlling the rate of fatty acid entry into the mitochondria. J Clin Invest (1996) 98(10):2244–50. 10.1172/JCI119034 8941640 PMC507673

[81] Volek JS Freidenreich DJ Saenz C Kunces LJ Creighton BC Bartley JM Metabolic characteristics of keto-adapted ultra-endurance runners. Metabolism (2016) 65(3):100–10. 10.1016/j.metabol.2015.10.028 26892521

[82] Webster CC Noakes TD Chacko SK Swart J Kohn TA Smith JA. Gluconeogenesis during endurance exercise in cyclists habituated to a long-term low carbohydrate high-fat diet. J Physiol (2016) 594(15):4389–405. 10.1113/JP271934 26918583 PMC4967730

[83] Elliott KR Harmatz JS Zhao Y Greenblatt DJ Body size changes among National Collegiate Athletic Association New England Division III football players, 1956–2014: comparison with age-matched population controls. J Athl Train (2016) 51(5):373–81. 10.4085/1062-6050-51.5.14 27159189 PMC5013707

[84] Yamamoto JB Yamamoto BE Yamamoto PP Yamamoto LG. Epidemiology of college athlete sizes, 1950s to current. Res Sports Med (2008) 16(2):111–27. 10.1080/15438620802103320 18569945

[85] Hruby A Bulathsinhala L McKinnon CJ Hill OT Montain SJ Young AJ Body mass index at accession and incident cardiometabolic risk factors in US army soldiers, 2001–2011. PLoS One (2017) 12(1):e0170144 10.1371/journal.pone.0170144 28095509 PMC5241140

[86] Gasier HG Hughes LM Young CR Richardson AM. Comparison of body composition assessed by dual-energy X-ray absorptiometry and BMI in current and former US navy service members. PLoS One (2015) 10(7):e0132157. 10.1371/journal.pone.0132157 26197480 PMC4510134

[87] Barr S Wright J. Postprandial energy expenditure in whole-food and processed-food meals: implications for daily energy expenditure. Food Nutr Res (2010) 54(1):5144. 10.3402/fnr.v54i0.5144 20613890 PMC2897733

[88] Ladabaum U Mannalithara A Myer PA Singh G. Obesity, abdominal obesity, physical activity, and caloric intake in US adults: 1988 to 2010. Am J Med (2014) 127(8):717–27.e12. 10.1016/j.amjmed.2014.02.026 24631411 PMC4524881

[89] Malhotra A Noakes T Phinney S It is time to bust the myth of physical inactivity and obesity: you cannot outrun a bad diet. Br J Sports Med (2015) 49(15):967–8. 10.1136/bjsports-2015-09491 25904145

[90] Luke A Cooper RS Physical activity does not influence obesity risk: time to clarify the public health message. Int J Epidemiol (2013) 42(6):1831–6. 10.1093/ije/dyt159 24415616

[91] Swinburn B Sacks G Ravussin E. Increased food energy supply is more than sufficient to explain the US epidemic of obesity. Am J Clin Nutr (2009) 90(6):1453–6. 10.3945/ajcn.2009.28595 19828708

[92] Basu S Yoffe P Hills N Lustig RH. The relationship of sugar to population-level diabetes prevalence: an econometric analysis of repeated cross-sectional data. PLoS One (2013) 8(2):e57873. 10.1371/journal.pone.0057873 23460912 PMC3584048

[93] Pfinder M Katikireddi SV Pega F Gartlehner G Fenton C Griebler U Taxation of unprocessed sugar or sugar-added foods for reducing their consumption and preventing obesity or other adverse health outcomes. Cochrane Libr (2016). 10.1002/14651858.CD012333 PMC7141932 32270494

[94] Hu FB Resolved: there is sufficient scientific evidence that decreasing sugar-sweetened beverage consumption will reduce the prevalence of obesity and obesity-related diseases. Obes Rev (2013) 14(8):606–19. 10.1111/obr.12040 23763695 PMC5325726

[95] de Ruyter JC Olthof MR Seidell JC Katan MB A trial of sugar-free or sugar-sweetened beverages and body weight in children. N Engl J Med (2012) 367(15):1397–406. 10.1056/NEJMoa1203034 22998340

[96] Ebbeling CB Feldman HA Chomitz VR Antonelli TA Gortmaker SL Osganian SK A randomized trial of sugar-sweetened beverages and adolescent body weight. N Engl J Med (2012) 367(15):1407–16. 10.1056/NEJMoa1203388 22998339 PMC3494993

[97] Qi Q Chu AY Kang JH Jensen MK Curhan GC Pasquale LR Sugar-sweetened beverages and genetic risk of obesity. N Engl J Med (2012) 367(15):1387–96. 10.1056/NEJMoa1203039 22998338 PMC3518794

[98] Maersk M Belza A Stødkilde-Jørgensen H Ringgaard S Chabanova E Thomsen H Sucrose-sweetened beverages increase fat storage in the liver, muscle, and visceral fat depot: a 6-mo randomized intervention study. Am J Clin Nutr (2012) 95(2):283–9. 10.1038/482027a 22205311

[99] Malik VS Popkin BM Bray GA Després J-P Hu FB Sugar-sweetened beverages, obesity, type 2 diabetes mellitus, and cardiovascular disease risk. Circulation (2010) 121(11):1356–64. 10.1161/CIRCULATIONAHA.109.876185 20308626 PMC2862465

[100] Caprio S Calories from soft drinks—do they matter. N Engl J Med (2012) 367(15):1462–3. 10.1056/NEJMe1209884 22998341

[101] Dietary Guidelines Advisory Committee. Advisory Report to the Secretary of Health and Human Services and the Secretary of Agriculture. (2015). Available from: https://health.gov/dietaryguidelines/2015-scientific-report/pdfs/scientific-report-of-the-2015-dietary-guidelines-advisory-committee.pdf

[102] Stanhope KL Schwarz J-M Havel PJ Adverse metabolic effects of dietary fructose: results from recent epidemiological, clinical, and mechanistic studies. Curr Opin Lipidol (2013) 24(3):198 10.1097/MOL.0b013e3283613bca 23594708 PMC4251462

[103] Yki-Järvinen H. Non-alcoholic fatty liver disease as a cause and a consequence of metabolic syndrome. Lancet Diabetes Endocrinol (2014) 2(11):901–10. 10.1016/S2213-8587(14)70032-4 24731669

[104] Slyper AH The influence of carbohydrate quality on cardiovascular disease, the metabolic syndrome, type 2 diabetes, and obesity – an overview. J Pediatr Endocrinol Metab (2013) 26(7–8):617–29. 10.1515/jpem-2012-0419 23729611

[105] Johnson RK Appel LJ Brands M Howard BV Lefevre M Lustig RH Dietary sugars intake and cardiovascular health: a scientific statement from the American Heart Association. Circulation (2009) 120(11):1011–20. 10.1161/CIRCULATIONAHA.109.192627 19704096

[106] Te Morenga L Mallard S Mann J Dietary sugars and body weight: systematic review and meta-analyses of randomised controlled trials and cohort studies. BMJ (2013) 346:e7492 10.1136/bmj.e7492 23321486

[107] Mennella JA Ontogeny of taste preferences: basic biology and implications for health. Am J Clin Nutr (2014) 99(3):704S–11S. 10.3945/ajcn.113.067694 24452237 PMC3927698

[108] Goran MI Dumke K Bouret SG Kayser B Walker RW Blumberg B. The obesogenic effect of high fructose exposure during early development. Nat Rev Endocrinol (2013) 9(8):494–500. 10.1038/nrendo.2013.108 23732284 PMC4916951

[109] Walker RW Goran MI. Laboratory determined sugar content and composition of commercial infant formulas, baby foods and common grocery items targeted to children. Nutrients (2015) 7(7):5850–67. 10.3390/nu7075254 26193309 PMC4517031

[110] Lustig RH Mulligan K Noworolski SM Tai VW Wen MJ Erkin-Cakmak A Isocaloric fructose restriction and metabolic improvement in children with obesity and metabolic syndrome. Obesity (2016) 24(2):453–60. 10.1002/oby.21371 26499447 PMC4736733

[111] Schwarz J-M Noworolski SM Erkin-Cakmak A Korn NJ Wen MJ Tai VW Effects of dietary fructose restriction on liver fat, de novo lipogenesis, and insulin kinetics in children with obesity. Gastroenterology (2017) 153:743–52. 10.1053/j.gastro.2017.05.043 28579536 PMC5813289

